# High quality statistical shape modelling of the human nasal cavity and applications

**DOI:** 10.1098/rsos.181558

**Published:** 2018-12-19

**Authors:** William Keustermans, Toon Huysmans, Femke Danckaers, Andrzej Zarowski, Bert Schmelzer, Jan Sijbers, Joris J. J. Dirckx

**Affiliations:** 1 Physics Department, University of Antwerp, Laboratory of Biophysics and Biomedical Physics, Groenenborgerlaan 171, 2020 Antwerp, Belgium; 2 Faculty of Industrial Design Engineering, TU Delft, Landbergstraat 15, 2628 CE Delft, The Netherlands; 3 Physics Department, University of Antwerp, Imec-Vision Lab, Edegemsesteenweg 200-240, 2610 Antwerp, Belgium; 4 ENT Department, GZA Sint-Augustinus Hospital, Oosterveldlaan 24, 2610 Antwerp, Belgium; 5 ENT Department, ZNA Middelheim Hospital, Lindendreef 1, 2020 Antwerp, Belgium

**Keywords:** nose, cylindrical parametrization, geometry average, morphology, age, gender

## Abstract

The human nose is a complex organ that shows large morphological variations and has many important functions. However, the relation between shape and function is not yet fully understood. In this work, we present a high quality statistical shape model of the human nose based on clinical CT data of 46 patients. A technique based on cylindrical parametrization was used to create a correspondence between the nasal shapes of the population. Applying principal component analysis on these corresponded nasal cavities resulted in an average nasal geometry and geometrical variations, known as principal components, present in the population with a high precision. The analysis led to 46 principal components, which account for 95% of the total geometrical variation captured. These variations are first discussed qualitatively, and the effect on the average nasal shape of the first five principal components is visualized. Hereafter, by using this statistical shape model, two application examples that lead to quantitative data are shown: nasal shape in function of age and gender, and a morphometric analysis of different anatomical regions. Shape models, as the one presented here, can help to get a better understanding of nasal shape and variation, and their relationship with demographic data.

## 1. Introduction

The human nose is an important and complex organ having many functions, including ventilation, filtration and olfaction. People who suffer from a nasal function impairment have a reduced overall quality of life [1]. Improving our knowledge of the key structures and key features of the nasal cavity is therefore important. The human nose shows large variation in shape between different individuals, for example variations of the bone structure [2,3] or temporal variations in the nasal cycle [4]. These variations alter the airflow through the nasal cavity, and can thereby affect the functioning of the nose [5]. To get a better understanding of normal and pathological functioning of the nasal cavity, it is important to study the effects of these variations. Nowadays, surface models of the nasal cavity are typically based on tomographic data coming from computed tomography (CT) scans. A whole range of studies have been conducted in the past, using data coming from a single or few patients [6,7], to studies where data of a much larger population is used [8–13]. The approach in this work is different from the previous in that it is not directly limited to the shapes obtained from the tomographic data. From this point of view, it is much more related to the work done by Gambaruto *et al.* [14] about nasal airflow modelling using the representation of a nasal passage based on a Fourier descriptor.

We propose to use the statistical shape modelling (SSM) technique to capture the shape variations [15]. In this way, we avoid *a priori* assumptions about morphology. SSM is a widely used tool to capture the morphological variability present in a population. A main application is their use as prior knowledge in model-based automated image segmentation. They were popularized for this purpose by Cootes *et al*. [16]. An overview of their use in medical image segmentation can also be found in Heimann & Meinzer [17]. In the present work, clinical CT data is used to capture the nose in 46 patients, and from this data a high quality statistical shape model is created. In literature, different techniques (e.g. level sets, Fourier descriptors) have already been used to create a shape model of the nose [18–22]. In Liu *et al.* [20] and Sun *et al.* [22] image alignment and image processing of cross-sections are used to create a standardized geometrical model. In the latter work, a full upper respiratory airway model is created, containing less details of the nasal complex. A similar approach is followed in Nejati *et al.* [21], where cross-sections of the segmented mesh are taken and processed. Thinned representations of the nasal cavity are then computed, and a reference template is deformed such that it matches this thinned representation. Finally, all deformations are averaged to obtain an average geometry. They reported an increase in tolerance to a wider variety of nasal geometries than earlier methods. However, none of the methods discussed above use the direct three-dimensional information coming from training shapes. In Huang *et al.* [19] level sets are used to construct a shape model. Their model and the shape instances it produces for different positions along the PC axes show artefacts, i.e. left and right nasal channels are fused at the top of the nasal cavity, and different turbinates are merged together.

The technique used in the present work, based on cylindrical parametrization, is highly suitable for creating a nasal shape model because of the shape of the nasal cavity. More precisely, all nasal shapes of the population were closed at the post-nasal region in such a way that the resulting nasal cavity can be seen as a U-bended cylinder. For more information, the reader is referred to §2.2. We will show that this approach not only allows capturing and qualitatively reporting the natural anatomical variations present, but also more importantly, enables to relate shape variations with other features. In the following, this is called the quantitative analysis of the nasal cavity. In this paper, we show two quantitative applications of the SSM: we look at how age and gender are related with nasal shape variations, and we apply the shape model to study and discuss the influence of shape variation on the volume, surface and surface-to-volume ratio (SVR) of certain anatomical regions. These regions are the nasal valve and nasal vestibule region, the respiratory region, the olfactory region and the post-nasal region. The anatomy of these regions is important for the well-functioning of the nose, e.g. respiratory region for heating and humidifying the inhaled air. For sake of clarity, it is defined what is meant with ‘shape’ in this work. Strictly speaking, when two objects fall perfectly together after alignment (due to rotation and translation), they are said to have the same ‘form’. When these two objects are additionally allowed to have a different relative scale, they are said to have the same ‘shape’. In this work, however, we consistently use the term ‘shape’ instead of ‘form’, due to its connotation with ‘statistical shape model’, although we did not correct for scale in the alignment.

## 2. Material and methods

### 2.1. Data acquisition

Clinical cone-beam CT (CBCT) scans of six patients and helical CT scans of 40 patients were analysed. Data were obtained from patients with very different nasal or sinus related complaints, where a CT scan was requested by the treating ear nose throat (ENT) physician. The spatial resolution of the scans ranges from 250 to 430 µm perpendicular to the axial scan direction, and from 250 to 600 µm in the axial direction. The group included 29 females and 17 males. The average male subject age was 37 years (minimum age is 17, maximum is 60), and the average female subject age was 46 years (minimum age is 20, maximum is 84).

### 2.2. Individual model generation

**Figure 1.**
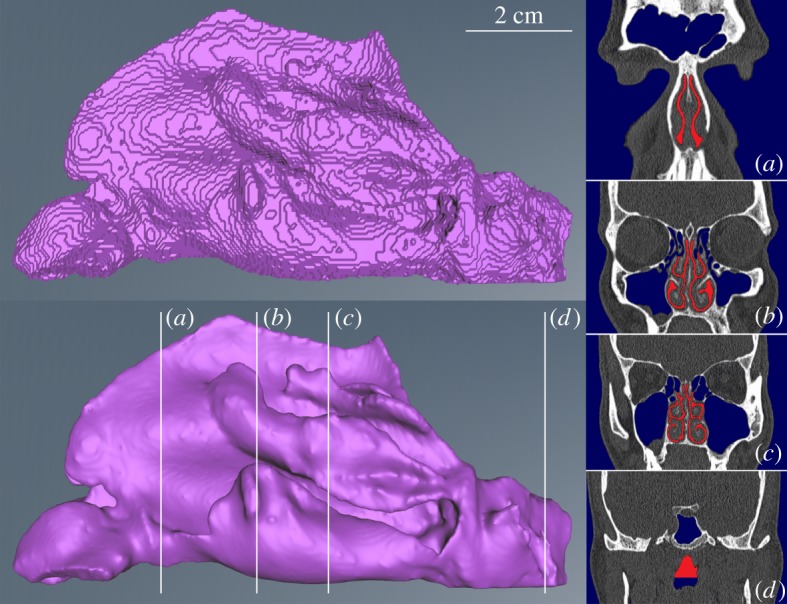
Segmentation result for a nose (side-view). Left top and bottom figures show the raw and smoothed nasal shape, respectively. The position of the four coronal slices (*a*–*d*) on the right are indicated on the shape. The applied mask is shown in blue, and the segmentation labels in red.

First, the different steps are given that were followed to generate 46 different surfaces of the nasal cavity based on CT data. A classical and well-known approach was used in this work. For each CT scan a three-dimensional geometrical model was built by segmentation using Amira v. 6.0.1. On CT slices of the nose one can observe air, soft tissue and bone. Greyscale thresholding was used to determine the nasal passages, because it is the simplest way of segmentation and very suitable to find air spaces in the skull. This, however, led to inclusion of the sinuses, which are connected to the nasal cavity. Manual separation of these sinuses was overseen by an ENT physician. This separation, and by extension all steps described below, was done by the same operator for all 46 CT scans to avoid inter-operator generated variations between segmentations. At this stage and where necessary, some small corrections were made in the file containing the result of the segmentation, from now on called ‘label file’. Eventually, the goal is to represent each nasal shape with the same amount of points, where those points are located at the same anatomical position. This is done in the surface correspondence step, discussed below, but demands that all the training surfaces have the topology of a sphere (genus-0). To meet this requirement, holes in the label files were therefore filled such that the eventual geometrical surface extracted from the label file is genus-0. The field of view also varies for each scan, so that the end of the post-nasal region is different for each nasal shape. For this reason, the labels cannot be used as is. This would introduce a large non-anatomical variation in the shape model, as variation would be the extent of the region over which the scan was taken. Therefore, the labels were cut perpendicular to the axial scan direction at the floor of the inferior turbinates (figure 1).

The generated labels are used to build a triangulated surface geometry by applying the marching cubes algorithm [23]. As a next step, these surface models were smoothed, taking care that potential surface shrinkage was minimal. Algorithms exist that can be used for this (for example, the surface smoothing algorithm in Amira v. 6.0.1 is based on the paper ‘Curve and surface smoothing without shrinkage’ by Taubin [24]). At the end, the surface models were still quite rough when, for example, comparing them to those used in computational fluid dynamics. Nonetheless, further smoothing was unnecessary because of the smoothing effect of averaging when deriving shape model instances. The number of triangles was not decimated, because this is not necessary for the correspondence procedure.

All surfaces were exported in the stereo lithographic (STL) file format, and tiny holes were cut in both nostrils at the same anatomical position for all shapes. This was the final prerequisite of the correspondence algorithm. To increase the amount of morphological variation captured by the statistical shape model, mirrored versions of all nasal cavities are generated. Paraview v. 5.4.0 was used to generate these mirrored shapes, hereby doubling the size of the training data for the statistical shape model. The average shape obtained from principal component analysis (PCA) will be symmetrical because of this step.

### 2.3. Surface correspondence

Surface correspondence defines a dense one-to-one map between the surfaces in the population. In this paper, the correspondence method of Huysmans *et al.* [15] was modified to guarantee a uniform surface sampling. The method proceeds in four main steps. In the first step, each surface was cylindrically parametrized, using the method of Huysmans *et al.* [25], equipping the vertices of each surface with u,v-coordinates. These coordinates define a low-distortion one-to-one mapping between each surface and the cylinder, where the small holes in the nostrils correspond to the two boundaries (ends) of the cylinder and where distortion is measured using the stretch metric of Sander *et al.* [26]. A correspondence between any two nasal cavities in the population can be derived by composition of these maps. In the second step, the surfaces were rigidly transformed and the parametrizations were rotated (u-translation) for optimal alignment according to the minimum description length (MDL) objective [27]. The MDL is a measure based on the minimum description length principle: the sampled surfaces are coded in a message where the encoding is determined by the PCA model built from the correspondence. The length of the total message together with the encoded model, determines the quality of the model, and in this way the quality of the correspondence. Because of this, a trade-off exists between goodness-of-fit and model complexity. In this work the simplified MDL measure that was introduced by Thodberg [28], is used,

2.1 $$\mu (\lambda_{1},\ldots ,\lambda_{n_{s}-1})=\underset{\lambda_{i}\geq \lambda_{c}\;\;}{\sum }\left(1+\log\frac{\lambda_{i}}{\lambda_{c}}\;\right)+\underset{\lambda_{i}<\lambda_{c}\;\;}{\sum }\frac{\lambda_{i}}{\lambda_{c}}\;\;.$$

**Figure 2.**
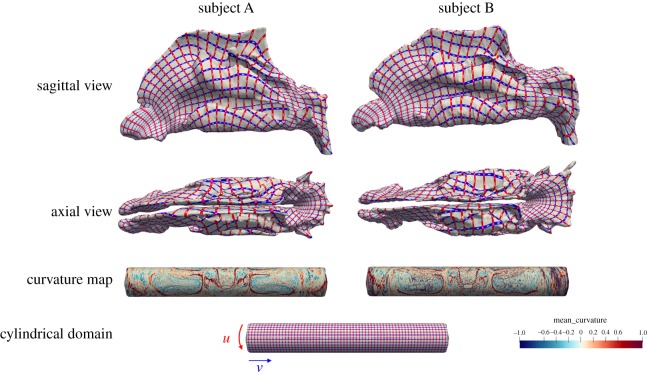
A visualization of the correspondence for two subjects (A and B). The top two rows show the sagittal and axial view of the nasal cavity surface where the red and blue lines reveal the (*u*,*v*) coordinates of the map to the cylinder. The red lines are at constant *v* (around the cylinder) and the blue lines at constant *u* (along the cylinder). The lines are at corresponding locations between A and B. Third row: the cylinder domain is colour coded with the mean curvature of the corresponding surface points, revealing the correspondence to the surface on the cylinder. The red lines correspond to the ridges of the post-nasal region (centre of the cylinder) and the turbinates (towards both sides of the cylinder). A very similar map is obtained for A and B indicating a good correspondence. Bottom row: the cylinder domain with *u* and *v* iso-parameter lines. These lines correspond to the lines on the nasal cavity surfaces.

It is a function of the shape mode variances $\lambda_{j}$. The parameter $\lambda_{c}$ is set to be the expected variance of the noise in the data, such that the variation captured by all modes with a variation smaller than $\lambda_{c}$ is considered noise. The MDL favours compact models, this can be seen from the fact that μ goes to zero when all eigenvalues go to zero, where a lower value of μ indicates a better quality of correspondence. In the third step, the correspondences were further fine-tuned by additional non-rigid deformations in the u,v-domain for each of the surfaces, measuring optimality again with the MDL objective. This optimization was done at three levels of detail. In the fourth and final step, the optimal parametrizations were used to generate a vertex correspondence for the population, where each shape is represented with a fixed number of landmarks that are at corresponding locations across the population. The term ‘landmarks’ here refers to Type III landmarks, defined by Bookstein [29] as mathematically derived points, which are different from Type I and II landmarks that are points at clearly identifiable features of the anatomy. The obtained correspondence is termed an anatomical correspondence in literature on image registration. This term is debatable, especially in inter-subject correspondences, where large differences in anatomy could be present. The obtained correspondence in this paper is the one that is optimal with respect to the MDL objective, commonly used in statistical shape modelling. As pointed out, the MDL objective measures the compactness of the PCA model calculated from the correspondences. Lower MDL values are assigned to correspondences that result in models with less variance. In this sense, models that only model variation in shape and do not include artificial variations like sliding of vertices along the surface are favoured. As it turns out, correspondences with optimal MDL tend to match up similarly shaped surface regions across the population, which are often also the same anatomical structures. See figure 2 for a visualization of the correspondence and cylinder domain for two subjects.

### 2.4. Statistical shape modelling

Using the correspondence calculated in the previous step, each shape is now represented by a set of 100 K corresponding landmark points distributed over the boundary surface of the nasal cavity and part of the post-nasal cavity. A SSM was built from the 92 corresponded nasal shapes by applying PCA on the vertices of the shapes. From a mathematical standpoint, a three-dimensional shape with *n* landmarks $\left(x_{i},y_{i},z_{i}\right)$ can be represented as a vector **x** with length 3*n*,

2.2 $$x={\left(x_{1},\ldots ,x_{n},y_{1},\ldots ,y_{n},z_{1},\ldots z_{n}\right)}^{T}.$$

For all 46 training examples and 46 mirrored counterparts, such a vector was generated. An important step is to ensure that all the shapes are optimally aligned. A nasal shape was considered to be independent of position and orientation, but not of scale. To determine the optimal poses, the Procrustes analysis was used. In this analysis, all shapes are aligned with a reference shape. In the first iteration no average shape is yet calculated, and cannot be used as reference shape. For this reason, a shape from the training set is chosen as reference shape to start the analysis. Because no scaling had to be used, the analysis came down to two steps. In the first part, differences due to translation were removed by calculating the centroid of each shape and translating them to the origin. In the second part, quaternions were used to compute the rotation matrix between each shape of the training set and the reference shape.

In a next step, PCA was applied, delivering an average nasal shape $\bar{x}$ (by averaging the corresponding landmarks) and an orthogonal set of shape variations (the so-called shape modes). Existing and new nasal shapes can then be described as the sum of the average nasal cavity and a specific linear combination of the shape modes. More formally, this is done by adapting the shape parameters b, so that the point coordinates will be displaced and a new surface x is created,

2.3 $$x\approx \bar{x}+Pb$$

and

2.4 $$b=P^{T}(x-\bar{x}).$$

***P*** contains the eigenvectors of the covariance matrix and ***b*** is a vector given by equation (2.4). It is common to limit the possible value of $b_{i}$ to the range ±3 times the standard deviation $\sqrt{\lambda_{i}}$, so that the model generates shapes similar to those in the training set. As most of the variance is concentrated in the first few shape modes, in general SSM can provide a compact parametrization of the space of possible shapes. This is dependent, among other things, on the shape under consideration and the amount of training data.

**Figure 3.**
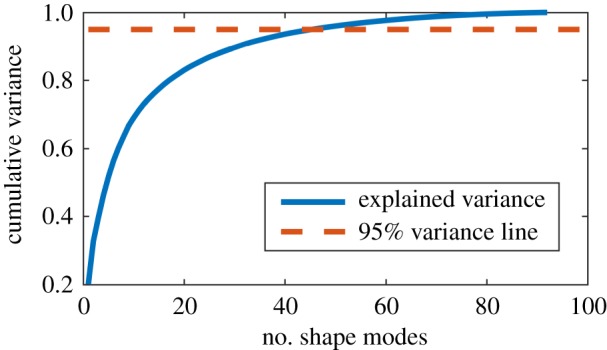
Cumulative variance of the principal components of variation (compactness). The dashed line indicates the 95% limit and intersects with the cumulative curve when the number of shape modes is equal to 46.

In figure 3, the normalized cumulative sum of all $\lambda_{i}$ is visualized. In the beginning the curve shows a strong increase, which indicates that these shape modes are important to include in the model. More to the right, the curve flattens and adding new shape modes to the model will add very little new variance, or unwanted variance caused by noise on the original data. A (95%) cut-off has to be chosen, and the variance that is captured by the resulting model is called ‘explained variance’ in figure 3.

### 2.5. Statistical analysis of shape

Because 95% of the total variation captured by the shape model is described by 46 shape modes, only these modes are considered in the following. This allows expressing the shapes of the training population in a useful way for further statistical analysis. More specifically, there are 46 values for each$\;b_{i}$, with *i* running from 1 to 46. It is purely coincidence that the amount of shapes of the training population is equal to the cut-off number for the parameters $b_{i}$; there is no mathematical constrict enforcing this.

The total population was divided according to gender. Next, for each parameter $b_{i}$ a simple linear regression model to age was created using R v. 3.4.2. This meant that in the end 46 simple regression models are obtained relating a specific $b_{i}$ with age. Normality was checked using a graphical technique called ‘normal probability plot’. In such a plot, the values for a parameter $b_{i}$ are plotted against age, and deviations from a straight line suggest deviation from normality. In case of deviation, a Box–Cox power transformation was applied. This is a procedure to find an appropriate value for the exponent *λ* to use to transform the original data into normal distributed data. The value of *λ* represents the power to which all data should be raised. To accomplish this, the Box–Cox power transformation searches for the best value for *λ* in a certain predefined region. The Box–Cox transformation function that was used has the form:

2.5 $$X_{t}(\lambda )=\{\begin{array}{cc} \frac{X_{o}^{\lambda }-1}{\lambda },\; & \lambda \neq 0\;\\ \log(X_{o}),\; & \lambda =0\; \end{array}$$

where $X_{t}$ and $X_{o}$ are the transformed and the original response variable, respectively. Outlier detection was based on Cook's distance, which measures for each data point the change of the regression fit with and without the presence of that specific point. In this way, the impact of each individual data point can be assessed and outliers can be detected. Robust regression is applied in the case of an outlier where the effect could not be neglected. The *t*-value coming from this robust regression is used to determine if the null hypothesis can be rejected or not (correlation or not). All regression models were tested at a 5% significance level.

This allows creating a visual map of how the shape of the nasal cavity is correlated with age. For the sake of clarity only the regression models of the female nasal shapes will be considered; the procedure is identical for the male shapes. As input, a lower age of 20, and an upper age of 80, is given to the regression model. The model outputs $\left\{b_{i}^{\mathrm{young}},b_{i}^{\mathrm{old}}\right\}$. For the models with *p* < 0.05, the corresponding entries are subtracted, giving $b_{i}^{\mathrm{diff}}$. The values $b_{i}$ that showed no significant correlation with age were set to zero. As a final step, one can calculate $x^{\mathrm{diff}}$ by using equation (2.3).

Other approaches similar to the one above exist, e.g. linear discriminant analysis, but for the sake of brevity we will not go deeper into them.

For the relation between nasal shape and gender a similar approach to the one above was used. For each corresponding male and female set of $b_{i}$, a two-sample *t*-test or a Wilcoxon test was used; the latter in case of non-normally distributed data. Significance was tested at a 5% level. Those values $b_{i}$ that showed no significant correlation with gender were set to zero, and $x^{\mathrm{diff}}$ was calculated.

### 2.6. Volume partitioning

**Figure 4.**
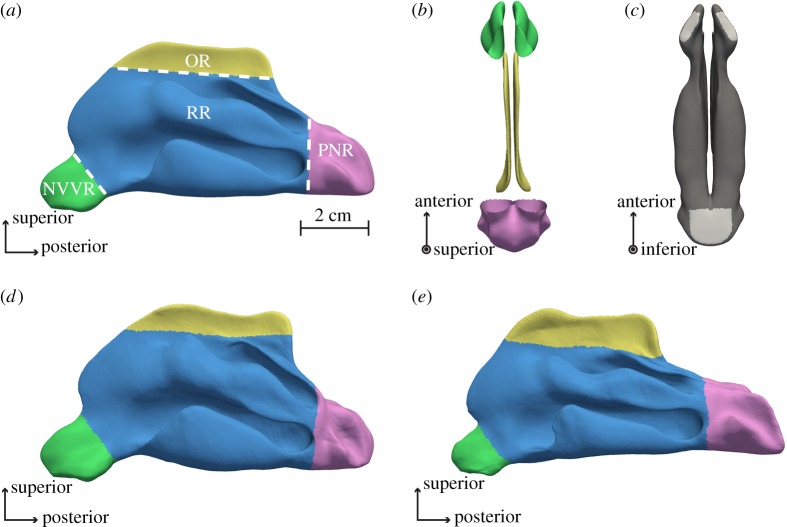
(*a*) The different anatomical regions: nasal valve and vestibule region (NVVR), olfactory region (OR), respiratory region (RR), post-nasal region (PNR). (*b*) Birds-eye view of nasal shape, where the RR is not visualized, showing the opening of the other regions. (*c*) Bottom view of the nose. In light grey, the regions that were subtracted from the NVVR and PNR are clearly visible. This subtraction is needed for a correct surface area calculation. (*d*,*e*) The result of the first principal component (PC) on the average shape with $b_{1}$ equal to $-2.5\sqrt{\lambda_{1}}$ and $2.5\sqrt{\lambda_{1}}$, respectively. It is clear that the regions defined before, vary accordingly.

A simple approach was followed to partition the total volume of each shape. The average nasal cavity was divided into four anatomical regions (figure 4*a*–*c*) based on [30–33]. The division was done using a bounding box in Paraview, and by selecting those triangular faces on the surface that comprise the different volumes of interest. This puts a constraint on the way the anatomical regions can be defined. A trade-off exists; lowering the box will at the same time add unwanted parts to the region. Therefore, the regions definition was overseen by an ENT physician and the current division made an optimal compromise. The nasal valve and nasal vestibule region compose a first region (green, NVVR). The respiratory region is shown in (blue, RR), the olfactory region on top in (yellow, OR) and posteriorly the post-nasal region in (pink, PNR). Because of the way the different regions are defined, the nasal valve (separating the NVVR and RR) is implicitly represented by a plane. In reality, the nasal valve has a non-planar shape. However, in literature it is not uncommon to make such an approximation because it allows a precise definition, e.g. for modelling purposes [32]. A fairly good agreement exists when comparing the anatomical regions with definitions in existing literature [31–33]. Small differences are not unusual because of a difference in nasal shape, and variation also exists between literature. For example, the definition of the olfactory region used in this work, has a better agreement with Croce [31] than with Netter [33]. As a last step the NVVR, OR and PNR were closed by defining a plane for each region connecting the boundary triangles, i.e. those triangles with neighbours at only one side. The tiny holes made for the correspondence step were also closed at this point. These steps made it possible to define a volume for all anatomical regions.

Finally, the volume of the respiratory region was calculated by subtracting the volume of the total nasal shape from that of the other three previously mentioned. The ten first shape modes are consecutively applied to the average shape with $b_{i}$ ranging from $-2.5\sqrt{\lambda_{i}}$ to $+2.5\sqrt{\lambda_{i}}$ in steps of $1.25\sqrt{\lambda_{i}}$ (see figure 4*d*,*e*). The effect of each shape mode on the surface area and on the volume of the different anatomical regions is captured and compared.

## 3. Results

### 3.1. Qualitative visualization

**Figure 5.**
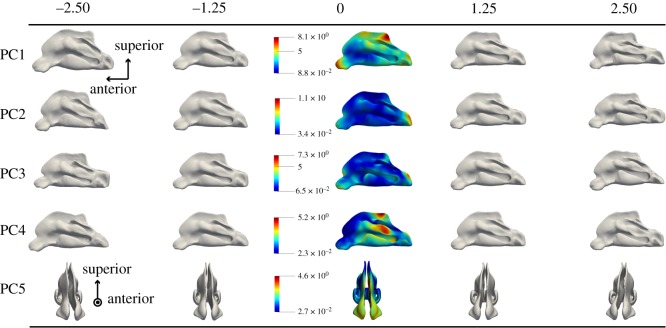
Results of applying the different PCs to the average shape, with different values for $b_{i}/\sqrt{\lambda_{i}}$. Each time the colour map represents the magnitude (in mm) of variation for $\text{P}{\text{C}}_{i}$.

The different shape modes can be visualized by applying them to the average nasal shape using equation (2.3). In figure 5 this is shown for the first five modes, with values for $b_{i}$ ranging from $-2.5\sqrt{\lambda_{i}}$ to $+2.5\sqrt{\lambda_{i}}$. For each PC, the colour map represents the magnitude of variation. Regular PCA is applied, which results in global modes, meaning that these modes act on the entire mean nasal surface.

The first eigenmode applies a general scaling to average nasal shape. It is not a perfect isotropic scaling, but nonetheless there is a clear size increase/decrease in all three dimensions. This could be expected because in the alignment of the shapes only rotation and translation were used and no scaling. The second mode predominantly results in the contraction or expansion of the nasal shape along the anterior–posterior axis, and can be clearly observed in the region behind the choana. The expansion of the shape along the anterior–posterior axis goes hand in hand with a, be it smaller, expansion in the perpendicular plane, making the nose wider. The third eigenmode has a large effect on the curvature of the line connecting the anterior tip of the nose and the end of the post-nasal region. More precisely: the anterior tip of the nose drops and the region behind the choana flattens with decreasing values for $b_{3}$. The fourth mode affects the width of the nasal shape. In addition, the posterior part of the olfactory region increases in height. It seems that the length of the olfactory region relative to that of the total nasal length is changed by this mode. The fifth eigenmode is the first one in line that has a non-negligible effect on the symmetry aspect of the nasal model. Applying this mode to the average nasal shape, which is symmetrical, results in bending the anterior part of the nose aside. One could say it reflects a specific type of nasal septum deviation. The fifth mode of the nasal shape is visualized from a front view, as the side view reveals little shape variation.

### 3.2. Quantitative applications

**Figure 6.**
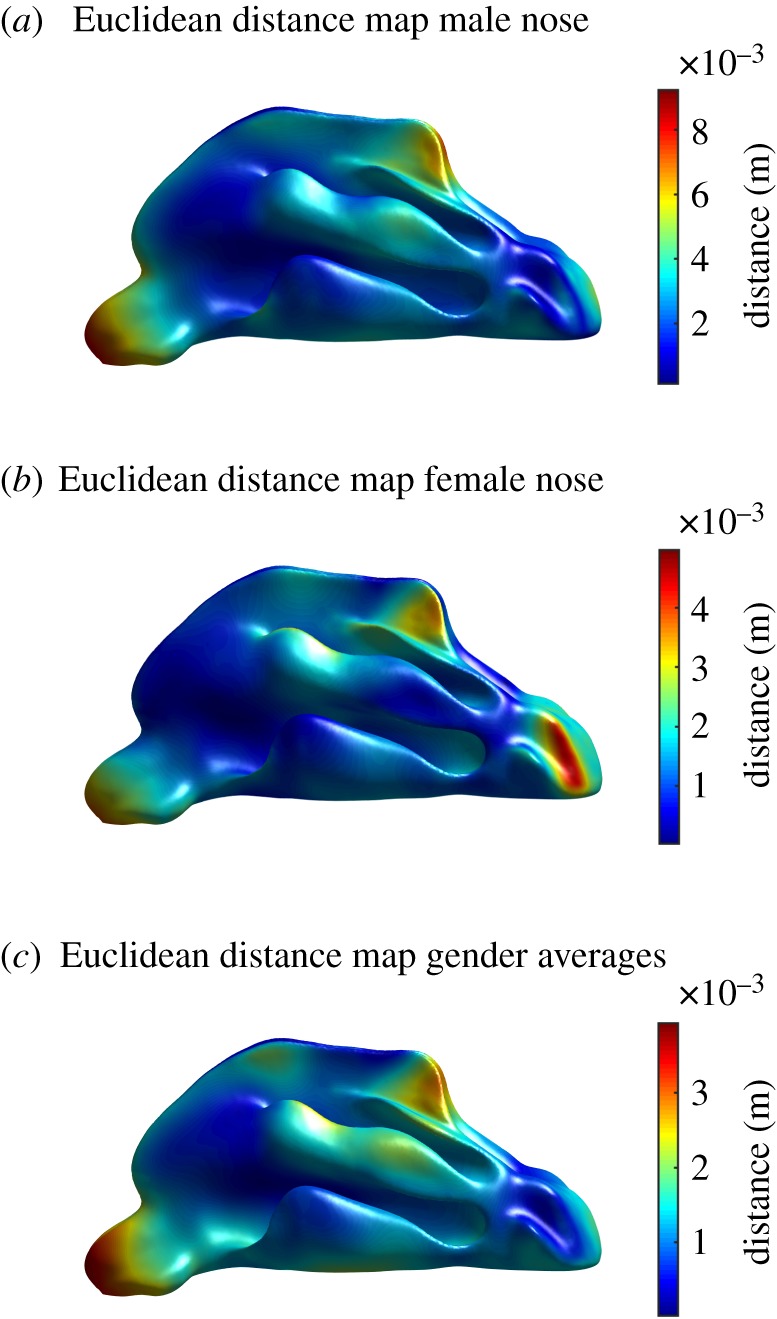
(*a*,*b*) Euclidean distance map between young and old nose, projected on the average nose, for males and females. These two maps show the magnitude and location of shape variation in function of age. (*c*) Euclidean distance map between average male and female nose, projected on the average nose. This map shows the magnitude and location of shape variation in function of gender.

Figure 6 shows the result of the statistical analysis described in the ‘Material and methods’ section. The analysis gave $\left\{b^{\mathrm{young}},b^{\mathrm{old}},b_{\mathrm{age}}^{\mathrm{diff}}\right\}$ and $\left\{b^{\mathrm{female}},b^{\mathrm{male}},b_{\mathrm{gender}}^{\mathrm{diff}}\right\}$ as a result. This is visualized by projecting the Euclidean distance map on the average nasal shape. This Euclidean distance $x^{\mathrm{diff}}$ is calculated from the vectors $b^{\mathrm{diff}}$ using equation (2.3). This allows creating a visual map of how the shape of the nasal cavity is correlated with age and gender.

### 3.3. Morphometric analysis of anatomical regions

#### 3.3.1. Nasal volume

As a second application example for the nasal shape model, the surface, volume and their ratio was studied for the different anatomical regions under the influence of each of the first 10 shape modes. These first 10 modes represent 69% of the total variation captured by the shape model. Not more are studied because of the extensive manual work involved per eigenmode.

**Figure 7.**
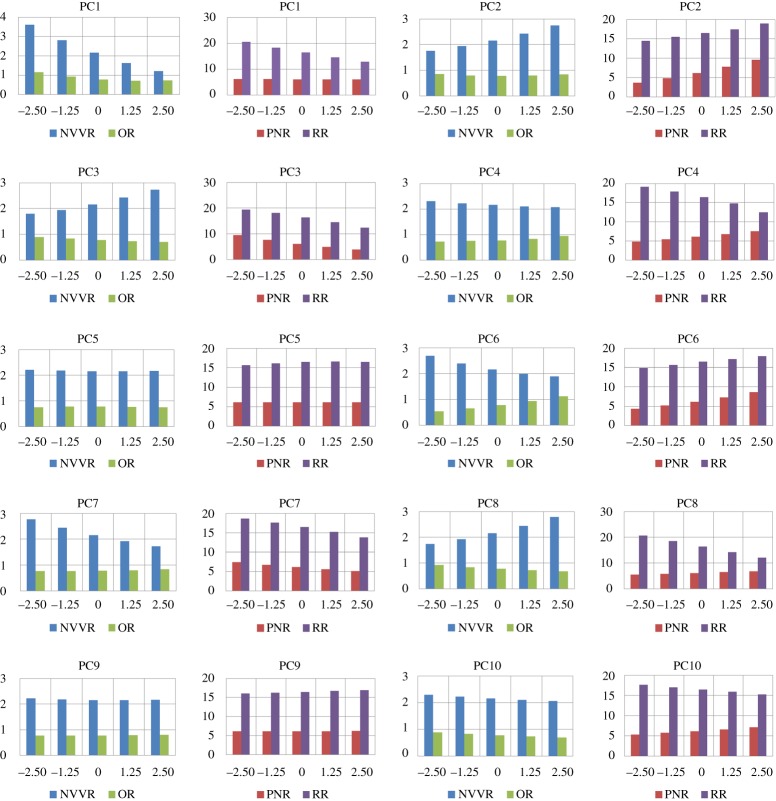
Bar chart for every principal component that is examined, indicating how the volume (in cm^3^ on the vertical axis) of each anatomical region changes with varying $b_{i}/\sqrt{\lambda_{i}}$ (on the horizontal axis).

The results of the analysis are summarized in figure 7. It consists of 20 charts, two for every shape mode. Each chart indicates how the volume, in cm^3^ on the vertical axis, of the anatomical regions changes for different values of the shape parameter $b_{i}$. The shape parameter is varied on the horizontal axis. The reason that there are two charts per shape mode is because of the large difference between the volumes of the NVVR and OR compared to that of the other two regions. Using one chart for all four regions would make the interpretation difficult. The different anatomical domains are abbreviated in the legend as before. The results of the analysis for some PCs are briefly described below. The values of the variation coefficient for the different regions can be found in table 1.

— PC1: has a negligible effect on the volume of the PNR, with a variance coefficient around 0.01. At the same time, PC1 definitely has an effect on the shape of the PNR that cannot be neglected. The volume of every other anatomical region reduces for $b_{1}$ going from $-2.5\;\sqrt{\lambda_{1}}$ to $2.5\;\sqrt{\lambda_{1}}$. The change in volume of the PNR sticks out with a value of 0.41.
— PC2: has a small effect on volume of OR. The volume of the other regions alters significantly.
— PC3: changes the volume in each anatomical region. Possibly interesting to notice is that the change in volume of the NVVR is inversely related to that of the other regions. When the volume of the others increases, that of the NVVR decreases.
— PC4: volume of the RR is inversely related to that of NR and OR. The NVVR has a variation coefficient 2.5–4 times smaller than the other regions.
— PC5: has a tiny effect on the volume of all regions, with 0.02 being the largest variance coefficient, belonging to the RR and OR. As stated in the qualitative description section, this is the first mode that results in an evident asymmetrical nature of the nasal cavity.
— PC8: while NVVR and PNR are increasing in volume, the opposite is happening for the OR and RR. The outer (anterior and posterior) volumes are thus demonstrating an inverse behaviour compared with the volume of the inner regions.
— PC9: second mode with an asymmetrical character. Just like for PC5, there is a negligible effect on all volumes.

**Table 1.** Volume-, surface- and SVR-variation coefficients (defined as the standard deviation divided by the mean) for the different anatomical regions and principal components.

	PC	1	2	3	4	5	6	7	8	9	10
volume	NVVR	0.41	0.18	0.17	0.04	0.01	0.14	0.19	0.19	0.01	0.04
	PNR	0.01	0.37	0.34	0.17	0.006	0.26	0.15	0.08	0.007	0.11
	OR	0.20	0.04	0.10	0.10	0.02	0.29	0.04	0.12	0.01	0.09
	RR	0.18	0.10	0.17	0.16	0.02	0.07	0.12	0.21	0.02	0.06
area	NVVR	0.30	0.12	0.12	0.006	0.01	0.05	0.09	0.13	0.01	0.05
	PNR	0.04	0.29	0.20	0.14	0.003	0.17	0.10	0.05	0.005	0.05
	OR	0.07	0.03	0.03	0.07	0.01	0.18	0.11	0.02	0.007	0.15
	RR	0.16	0.09	0.02	0.03	0.01	0.04	0.04	0.02	0.007	0.02
SVR	NVVR	0.12	0.06	0.05	0.04	0.005	0.09	0.10	0.06	0.003	0.01
	PNR	0.04	0.09	0.15	0.05	0.005	0.1	0.05	0.05	0.004	0.07
	OR	0.12	0.04	0.07	0.03	0.03	0.11	0.07	0.11	0.02	0.05
	RR	0.03	0.02	0.20	0.20	0.03	0.04	0.16	0.23	0.03	0.04

#### 3.3.2. Nasal surface

**Figure 8.**
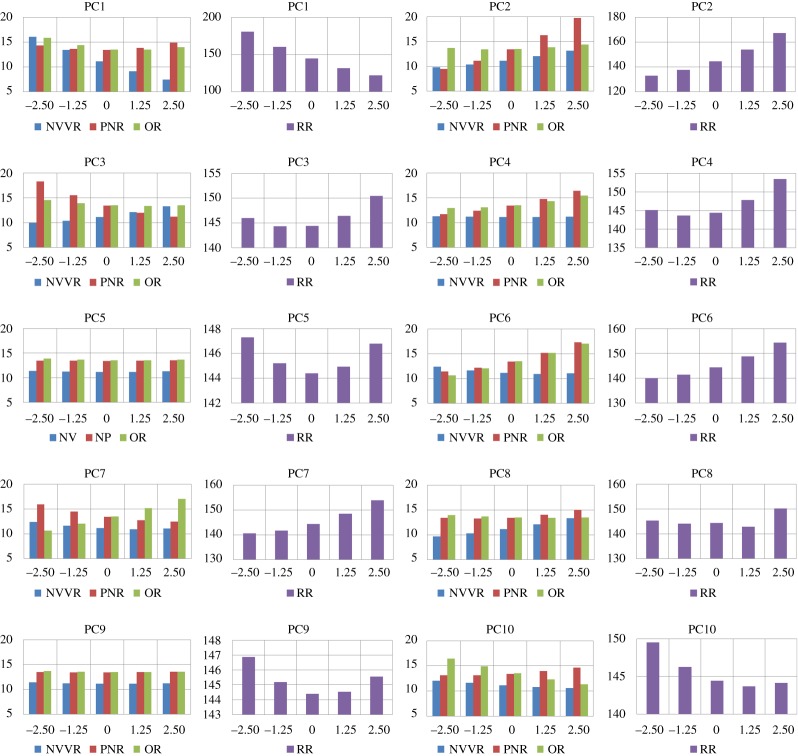
Bar chart for first 10 principal components, indicating how the surface area (in cm^2^ on the vertical axis) of each anatomical region changes with varying $b_{i}/\sqrt{\lambda_{i}}$ (on the horizontal axis).

The results of the analysis are summarized in figure 8, which consists of 20 charts: two for every shape mode. Because of the large difference between the surface area of the RR and that of the other three regions, these are visualized separately. Each chart indicates how the area, in cm^2^ on the vertical axis, of the anatomical regions changes for different values of the shape parameter $b_{i}$. The shape parameter is varied on the horizontal axis.

— PC1: has a rather small effect on the surface area of the PNR. The surface area of the other anatomical region decreases for $b_{1}$ going from $-2.5\;\sqrt{\lambda_{1}}$ to from $2.5\;\sqrt{\lambda_{1}}$. The first mode mostly affects the surface area of the NVVR.
— PC2: has a small effect on the surface area of the OR in comparison to that of the other regions, as can be seen from the variation coefficients.
— PC3: the variation in surface area of the outer regions (NVVR and PNR) is considerably larger than that of the OR and RR, where the latter is almost negligible.
— PC4: effect on surface area of NVVR is almost non-existent. The surface area of the PNR and OR increases for increasing values of $b_{4}$.
— PC5: the variation coefficient of every anatomical region is small. This mode has almost no effect on the surface area of the nasal cavity.
— PC7: the size of the surface area variation of every region except the RR is almost equal, with a variation coefficient around 0.1. The area of the OR has an opposite trend in comparison to the NVVR and PNR, decreasing when the other two regions are increasing.
— PC9: second mode with an asymmetrical character. There is a negligible effect on the surface areas. The highest value for the variation coefficient is for the NVVR, with a value of 0.01.

#### 3.3.3. Nasal surface area to volume ratio

**Figure 9.**
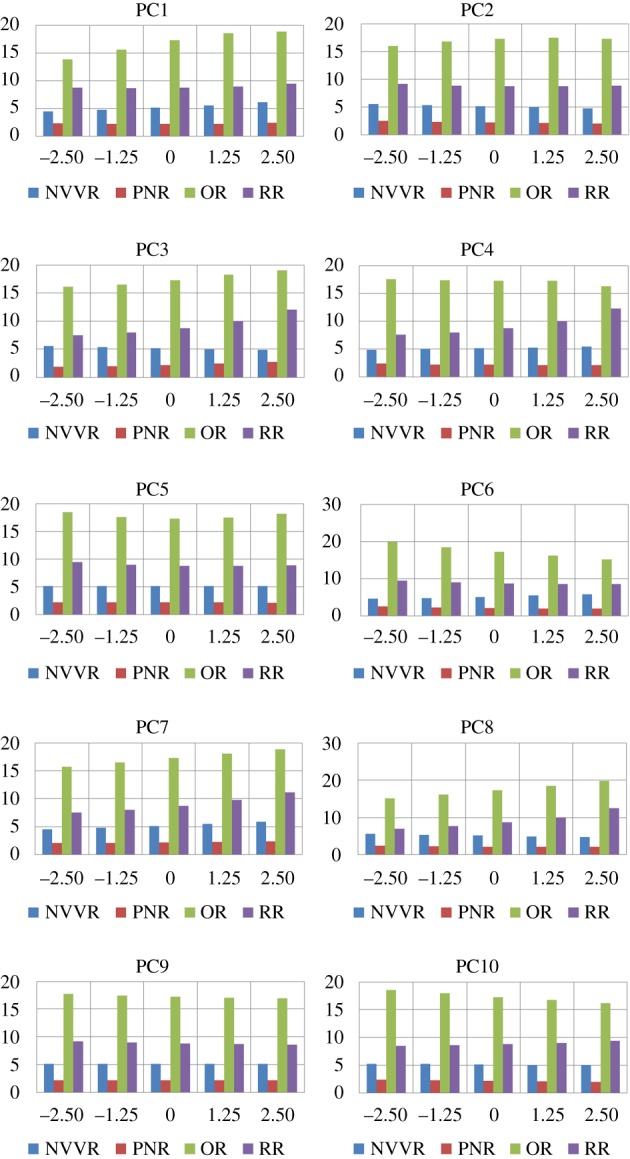
Bar chart for first 10 principal components, indicating how the SVR (in cm^−1^ on the vertical axis) of each anatomical region changes with varying $b_{i}/\sqrt{\lambda_{i}}$ (on the horizontal axis).

The results of the analysis are summarized in figure 9, which consists of 10 charts. Each chart indicates how the surface-to-volume-ratio (SVR), in cm^−1^ on the vertical axis, of the anatomical regions changes for different values of the shape parameter $b_{i}$. The shape parameter is varied on the horizontal axis.

— PC1: has a small effect on the SVR of PNR and RR. The variation coefficients of the NVVR and OR are almost equal, with a value around 0.12. For all regions, SVR increases with increasing $b_{1}$.
— PC3: is complementary to the first mode in the sense that most of the variation of SVR is located at the PNR and the RR, and not so much at NVVR and OR. The SVR of the NVVR changes inversely related to that of the other regions.
— PC5: the variation coefficient of every anatomical region is quite small, with the highest value of 0.03 for the RR and OR. This mode has almost no effect on the SVR of the nasal cavity.
— PC6: except for the RR, the variation for the other regions is almost the same. The SVR of the NVVR increases for increasing values for $b_{6}$, while for the PNR and OR it decreases.
— PC8: the values for SVR of the outer regions (NVVR and PNR) demonstrate an inverse behaviour in comparison to the inner regions.
— PC9: just like for PC5, here the variation of the SVR of every region is small. The highest value for the variation coefficient is for the RR, with a value of 0.03.

## 4. Discussion

### 4.1. Clinical CT data

The entire statistical analysis starts with tomographic data from patients, collected at the hospital. The scans have a non-isotropic voxel size and it was mentioned in §2.1 that the spatial resolution could differ between different scans. Because the nasal cavity is a complex structure, containing thin regions, one should be aware that CT as an imaging technique with finite resolution causes an uncertainty on the exact dimensions of the scanned nose and, as a direct consequence, on the exact dimensions of the geometrical surface model obtained through segmentation. Because of the non-isotropic voxel size of the scans used in this work, a larger voxel size in the axial direction did not automatically mean that the in-plane resolution was also lower. In this way, in every scan that was used, it was possible to identify the thin regions, such that they could be segmented. Nevertheless, as mentioned above, the accuracy is always limited by the tomographic imaging process. This uncertainty on the exact nasal dimensions is of the same order as that by choice of segmentation threshold.

In the present study, clinical CT and CBCT data were used to calculate the average morphology and their variation with a high precision. Because of the resolution constrictions of clinical imaging, the study is limited to ‘macroscopic’ (greater than 500 µm) shape variations. In the case of the nasal cavity, however, this poses no issues because the interesting shape variation is situated above this lower limit and thereby captured.

### 4.2. Statistical shape modelling

To create a SSM, surface models are needed. These surface models are created by segmentation, which is to some degree dependent on the operator's subjective input. In this study, all the segmentations are done by the same operator to avoid inter-operator generated variation in the shape model. In this way, unnatural shape model variation, resulting from segmentation differences between scans, is avoided as best as possible. Because regular PCA is used, global shape modes were obtained. Therefore, it is unrealistic that the influence of these segmentation differences expresses itself in the form of a new unnatural shape mode. In all probability, these differences have an influence on only a fraction of the natural modes of the nasal shape model. The modes calculated from PCA can be ranked in function of the size of their respective standard deviation. When care is taken in the segmentation process to avoid inter-operator induced segmentation variations, such unnatural variations should only affect natural shape modes with a small standard deviation (positioned in the far right side of the graph of figure 3). In this right side, one also finds the unnatural shape modes originating from CT data noise. One should be aware of this, and when almost all shape modes would be investigated, a cut-off value for the standard deviation should be defined. Only shape modes with a standard deviation higher than this cut-off value should be seen as representing natural variation. This cut-off value can be based on the standard deviation of the noise present in the CT data. In this work no cut-off value is calculated because only the first 10 modes, representing 69% of the total variation, are used.

In this study a combination of CT and CBCT images were used. One should be aware that small segmentation differences can therefore never be fully excluded. Not even when one operator performed all segmentations. The reason is that the segmentation threshold settings are different for CT and CBCT, which has an influence on the size of the model.

Because the training shapes of the statistical shape model were obtained by segmentation of tomographic data, this inherently delivers staircase-liked geometrical shapes as a first result. These staircases are unnatural, and smoothing was applied. Smoothing, however, can cause surface shrinkage. In this work we investigated nasal shape variation, and this variation can be influenced by surface shrinkage of the training data. This is the reason why these surface models were only mildly smoothed, taking care that potential surface shrinkage was minimal. This, however, does not mean that the surface of the shape modes, to which the real interest goes out in this work, is not smooth. The reason for this is the smoothing effect of averaging when shape modes are derived, as mentioned in §2.2. In view of the statistical analysis, where volume and surface data is calculated in function of the applied principal component, it is better to start with training data that have a somewhat rougher surface. Otherwise, the volume and surface area of the shape modes could be influenced by the surface shrinkage of the training data due to a too large smoothing.

Another source of subjective errors is the selection of the anatomical regions. When one would perform such a study on non-corresponded shapes, these anatomical regions would have to be manually indicated on every shape. This process is prone to error and very time consuming. In this study, we took advantage of the correspondence that exists between the average shapes and existing shapes/new shapes. It can be expected that the correctness for this approach is better than by selecting the regions on each shape, because of operator-induced errors, on the condition of a high quality correspondence between the training shapes. Here an advantage of SSM is the dense correspondence between different shapes. The reason is that all surface points are corresponded instead of only annotated landmark points. In this way, the relation between the parameters and the global shape can be visualized.

Alternative methods exist that allow the construction of correspondences, e.g. the slice-wise correspondence method of Gambaruto *et al.* [14]. Different approaches typically lead to different correspondences and derived SSMs. In this paper the method of Huysmans *et al.* [15] was chosen as it considers the full three-dimensional surface of the nasal cavity when optimizing correspondences. In contrast, the method of Gambaruto *et al.* does not take full advantage of having the three-dimensional nasal cavity surface available. In their method, the correspondence is established in 50 two-dimensional coronal slices spaced differently in the middle part versus the anterior and posterior part. There are several drawbacks of establishing a correspondence in-slice. First of all, out-of-slice correspondences are not possible, thereby ignoring natural variation in the anteroposterior direction. Secondly, they only model a single contour per cross-section. Slicing a three-dimensional surface can, however, result in multiple non-connected contours, namely when the surface goes in and out of the slice. The reduction to a single contour results in an unnatural flattened shape at the interface between the respiratory and post-nasal regions and the interface between the respiratory and nasal valve regions. This is noticeable in their average geometry. The method presented in this work retains a three-dimensional surface representation at all times and therefore does not suffer from these drawbacks.

Another major advantage of SSM is the small amount of parameters that are needed to describe the shape variation. Most of the large-scale variation can be represented with a limited number of parameters, because higher PCs only represent small variations. For the nasal shape, the combined contribution of PC 47–95 is around 5%. When using shape modelling to examine variation in flow as a function of variation in shape, care needs to be taken. Small variations in shape do not necessarily imply small influence on flow through the nose. Therefore, higher order PCs potentially have a non-negligible effect on the fluid mechanics.

A disadvantage of the SSM technique used in this work is that all shapes need to have the same topology. This is not the case for every SSM approach, e.g. level-set based SSMs. The topology of the nasal shape had to be the same as that of a sphere (genus-0), and this is not always correct for every nose, for example in the case of a nasal septum perforation. Problems like this can be detected, and can subsequently be manually dealt with.

In the near future, we will use this approach to study the influence of morphology on different flow characteristics of the nasal flow, e.g. nasal resistance, heat transfer, flow humidity. For such a study, it would be better to include an extra part at the back end of the nasal shape, at the post-nasal region. Having an average nasal shape will enable to study the flow, without being limited to data of a single patient. Next, geometrical variation can be studied by doing a parametric study including the mode shapes. In addition, a sensitivity analysis can determine the most important mode shapes for the proper functioning of the nose. Another possibility is to change certain features, in order to test their influence. This would have the most clinical value, because an ENT physician can relate far easier to anatomical features than to PCs. Finally, knowing the morphometric data of a population of healthy noses may be helpful for the classification of different nasal pathologies.

### 4.3. Quantitative analysis

The analyses that are discussed below are application examples of what can be done with a high quality SSM of the nasal cavity. The results in themselves are relevant, despite limited knowledge of patient's metadata, e.g. population composition, pathologies. A future analysis, using a larger sample size, should contain this information to be able to make substantiated conclusions about nasal shape relations. One can control for other factors, e.g. mucosal thickness, to ensure that the variation of the nasal cavity as exhibited by the shape model is driven by real morphological variation. In such an analysis, it would also be good to separate size, allowing the investigation of allometric and non-allometric contributions. Even more relevant are the applications, a standard nasal cavity model can for example be applied in the pharmaceutical industry (in the field of nasal drug delivery) [34,35], for disease diagnosis [36], or for toxicology [37,38]. Climate-related variation [3] or variation of the nasal shape due to ethnic descent could be examined by creating a SSM for every climate or ethnicity and comparing them in terms of their average and PCs. A SSM based on cylindrical parametrization can also contribute to other areas. For example, during stress and particle deposition surface mapping of the nasal wall during inhalation. By transforming the three-dimensional nasal cavity into a two-dimensional domain, obscured regions in the complex nasal geometry are revealed and wall shear stresses can be viewed on a two-dimensional map. In Inthavong *et al.* [39] surface cuts are introduced to obtain such a two-dimensional map, something which is not needed in case of a cylindrically parametrized model. Figure 6 shows the relation between nasal shape and respectively age and gender. It is noticeable that (*a*) and (*c*) give an almost equal result. The reason for this is that in both cases the first eigenmode $b_{1}$ showed a significant variation during the statistical analysis, in contrast to some successive eigenmodes (whose $b_{i}$ was set to zero). Because the first few modes capture most of the variance in the nasal shape model, this first mode has a bigger weight in the combined variation. A gender imbalance in the population for old versus young noses could be the origin, causing the main variation to relate also to gender.

A first noticeable result is that the fifth and ninth mode both have a negligible effect on the surface area, volume and (as a result) on the SVR of all four anatomical regions. When one inspects the effect of all 10 PCs on the symmetrical average nasal shape, it turns out that only the result of these two PCs is a clear asymmetrical shape. The shape model learned this variation from the total (original and mirrored) training set of noses. When looking at the asymmetrical modes five and nine, it is clear that an equal magnitude for $b_{i}$ (e.g. $-2\surd \lambda_{5}$ and $+2\surd \lambda_{5}$) results in shapes that are each other's mirror counterparts, and for mirrored shapes, there indeed exists no variation of volume and surface area. However, it turns out that for $b_{i}$ going from 0 to ±$2.5\surd \lambda_{i}$, the effect on the volume and surface area is also minimal. This cannot be assigned to the mirroring and therefore it can be argued that this is a general feature learned from the original noses.

For some eigenmodes, the shape model also showed an inverse relation between the volume of the nasal vestibule/nasal valve region and the respiratory region. This convergent-divergent ratio of nasal volume has an effect on the airflow, which might have a considerable effect on the amount of turbulence.

Important to mention, is that the order of the SVR is subjective to the chosen units (here centimetres), for example the SVR is one order higher when millimetres are used. This has to be kept in mind when comparing results.

One of the most important tasks of the respiratory region is to humidify and heat the incoming air, and recycle this heat and moisture during the expiration phase. One therefore could expect two things. First of all, the olfactory and respiratory region having the largest surface-to-volume ratio of the four anatomical regions, both for different reasons: the olfactory region, because particles have to have a large chance of hitting the nasal wall, to make olfaction possible; the respiratory region because it has an important task in heating and humidifying the incoming air. Second, one could expect that the variation of the SVR is more constrained for these regions (by nature), because it has a large impact on its functioning. This second concept was not confirmed by the statistical analysis. One of the reasons could be the current limitation: the training population was built from people who have a nasal airway or nasal sinus complaint. It could be that people had a complaint because of this, and so these nasal shapes are part of the training population. For this reason, not much can be concluded about relationships between volume and SVR of the nasal cavity and proper functioning (heating of air, and so on). The aim of the current work was to give an outlook of the chosen method. However, in the future it would be an ideal case to have a pathological shape model and a healthy shape model to do further research and be able to have substantial conclusions.

## 5. Conclusion

In this work, a high quality statistical shape model of the nasal cavity was built based on a population of nasal shapes. It was shown that such a high quality model can be used to examine the morphological variations exhibited by the underlying population, and relate nasal shape with other metadata such as age and gender. Some limitations of the current model put a limit on what can be concluded. In the future, the method will give the possibility to develop a pathological shape model and a healthy shape model. We would furthermore like to analyse the nasal cavity as a bilaterally symmetric object and separate symmetric and asymmetric variations. We will also use this approach to study the influence of morphology (e.g. symmetry or lack of it) on different characteristics of the nasal flow, e.g. nasal resistance, heat transfer, humidity and particle deposition.

## Data accessibility

Data are available from the Dryad Digital Repository: http://dx.doi.org/10.5061/dryad.75fq000 [40].
